#  Entre os muitos partos no Brasil, conhecer aqueles na luz do Daime

**DOI:** 10.1590/0102-311XPT104523

**Published:** 2023-09-18

**Authors:** Andreza Pereira Rodrigues

**Affiliations:** 1 Escola de Enfermagem Anna Nery, Universidade Federal do Rio de Janeiro, Rio de Janeiro, Brasil.

O parto e o nascimento são eventos que interessam, e muito, à sociedade, como já nos disse Maria Lúcia Mott ^1^. E, para serem compreendidos, precisam de múltiplos olhares. Diferentes disciplinas produzem aproximações distintas, que, somadas, ampliam a noção da complexidade desses eventos. Em que pese essa complexidade, nem sempre nos atentamos ao modo como ela se traduz nas práticas de atenção à saúde. O parto e o nascimento têm sido alvo de políticas públicas que buscam incidir em mudanças relevantes nas suas práticas, entre elas a realização excessiva de cirurgias cesáreas, em um contexto mais ampliado de medicalização ^2^. O cenário do parto no Brasil pôde ser melhor conhecido a partir do inquérito nacional sobre parto e nascimento (2011-2012) ^3^, mas cada nova abordagem nos apoia no percurso de desvendar especificidades desses eventos, de modo a garantir uma atenção pautada nos direitos (não só reprodutivos), na equidade e na humanização.

É nesse sentido de contribuição para a compreensão da diversidade e complexidade do parto que o livro *O Parto na Luz do Daime: Corpo e Reprodução entre Mulheres na Vila Irineu Serra*^4^é aqui apresentado. Fruto da tese de doutorado da autora Juliana Nicolle Rebelo Barretto, a etnografia de oito meses junto à comunidade hoasqueira da Vila Irineu Serra - localizada no Município de Rio Branco, Estado do Acre, Brasil - provoca reflexões sobre relações de poder e hierarquias de gênero, fronteiras entre remédio e drogas (discussão muito bem situada e argumentada, por sinal) e, o ponto chave, sobre corpo e reprodução entre mulheres que interseccionam os rituais de parto e os rituais religiosos.

Essa intersecção talvez seja uma grande novidade para os pesquisadores e leitores sobre gestação e parto no Brasil. Com riqueza de detalhes, é possível mergulhar com a autora na compreensão de como gestações, partos e pós-partos foram vivenciados na cultura do Daime no Acre por mulheres “hoasqueiras” - em referência a hoasca, ou ayahuasca, uma bebida feita geralmente a partir da infusão vegetal.

O livro, desde a apresentação, instiga a problematizar a convivência de saberes e os percursos de cuidados empreendidos por diferentes mulheres, a forma como “o sistema de saúde” lida com mulheres que conjugam esses saberes e o traduzem em seus rituais de parto e, sobretudo, como hierarquias de gênero são preservadas (ou subvertidas) pelo feminino reconhecido como força e potência na reprodução.

Os três primeiros capítulos situam o leitor de modo contundente no tema. O capítulo 1 - *Hoasca e as Políticas no Brasil* - é base fundamental, em especial para quem não tem proximidade com a discussão sobre ayahuasca e as disputas narrativas entre uso religioso e consumo nocivo de drogas alucinógenas, principalmente entre gestantes. Os capítulos 2 - *Pesquisa de Campo* - e 3 - *A Vila Irineu Serra* -, além do texto, são enriquecidos por desenhos e fotos, que permitem ao leitor conhecer como e onde a pesquisa foi realizada. Neles, a autora registra os passos da incursão etnográfica, o acolhimento na vila, sua participação nos encontros, além de mostrar como os desenhos podem ser ricos em registros de campo na pesquisa. A Vila Irineu Serra ganhou vida com texto e imagens, assim como com a descrição dos rituais, hinos e o detalhamento da relação da comunidade com Nossa Senhora da Conceição.

Os capítulos 4 - *Partos, Daime e Feminização da Hoasca* - e 5 - *Corpo e Percepção* - são o ponto alto do livro. Neles, vemos as relações de gênero no ritual religioso, que, com o parto - evento de mulheres -, ganha novos contornos, bem como a convivência entre religião e biomedicina, que ora são complementares e ora a primeira é soberana e exclui a outra. Apoiada de modo consistente em literatura específica da antropologia, a autora guia o leitor por um universo em que o parto desafia a lógica dos saberes biomédicos. Desse modo, conhecimentos da medicina e da religião transitam e não determinam uma forma única, nem de lugar - casa ou hospital -, nem de práticas - força do parto e força do Daime -, mas se interseccionam e produzem modos próprios de sentir, viver e compreender os eventos reprodutivos no corpo feminino.

O “*Daime que protege*” e “*a bebida que ajuda*” são a tônica do capítulo 5, em que vemos em minúcias como os eventos do corpo feminino são percebidos como canais abertos à potencialização pela bebida, assim como o ritual de recepção do filho na crença, com o nascimento à luz do Daime. Desses capítulos, fica a síntese de que a vivência na comunidade de Vila Irineu Serra no contexto atual e as transformações nas práticas de parto (internas e externas à comunidade) ressignificam as compreensões do que é um corpo feminino entre as mulheres hoasqueiras.

Fechando o livro, as considerações finais da autora são um convite a relembrar os pontos centrais e a sair dessa imersão no parto na luz do Daime levando boas reflexões.

O livro é interessante para quem gosta de literatura sobre parto, mas destaco também como necessário para os que apreciam o estudo de rituais. Para aqueles que acompanham discussões sobre o uso da “beberagem”, a leitura pode ser uma incursão relevante, considerando que o consumo da bebida nos rituais religiosos ainda é um dilema, por vezes incluído no contexto das políticas relacionadas às drogas. A presença da hoasca nos eventos de gestação e parto pode ser ainda mais desafiadora à compreensão, mas de certo subsidiará a compreensão do sagrado (feminino) proporcionado por ela.
